# Nutritional and inflammatory markers for predicting delirium after radical hysterectomy for cervical cancer: development of a nomogram

**DOI:** 10.3389/fnut.2025.1644157

**Published:** 2025-11-17

**Authors:** Yabin Zhu, Dong Xiang, Hailin Xing, Yunxiang Li, Hong Xie, Lin Jiang

**Affiliations:** 1 Department of Anesthesiology, The Affiliated Taizhou People's Hospital of Nanjing Medical University, Taizhou School of Clinical Medicine, Nanjing Medical University, Taizhou, Jiangsu, China; 2 Department of Anesthesiology, The Second Affiliated Hospital of Soochow University, Suzhou, China

**Keywords:** cervical cancer, postoperative delirium, risk factor, nutritional score, nomogram model

## Abstract

**Background:**

This investigation aimed to establish pivotal determinants of postoperative delirium (POD) following radical hysterectomy for cervical carcinoma (CC) and formulate an individualized risk stratification tool.

**Methods:**

We conducted a retrospective cohort study encompassing 253 geriatric patients undergoing radical hysterectomy for CC between 2021 and 2025. We systematically evaluated potential predictors using a two-phase regression model: first through univariate analysis (*P* < 0.1), followed by multivariate logistic regression (*P* < 0.05) to identify independent predictors of POD. Key clinical, demographic, and laboratory variables were included in the analysis. The incidence of POD was assessed using the Confusion Assessment Method (CAM) during the 7-day perioperative period. The predictive nomogram was developed using R and was rigorously validated through both internal cohort validation and external validation. ROC, calibration, and decision curve analyses were used to assess the nomogram's predictive performance.

**Results:**

The POD incidence reached 16.2% (*n* = 41) during the 7-day postoperative surveillance. Multivariable analysis delineated five independent predictors: advanced age (OR = 1.12, *P* = 0.031), depressed albumin-fibrinogen ratio (AFR; OR = 0.69, *P* = 0.029), elevated neutrophil-lymphocyte ratio (NLR; OR = 3.51, *P* = 0.001), Controlling Nutritional Status (CONUT) score (OR = 1.81, *P* = 0.003), and Geriatric Nutritional Risk Index (GNRI; OR = 0.94, *P* = 0.001). The constructed nomogram exhibited robust discriminative capacity, achieving area under curve (AUC) values of 0.821 and 0.966 in internal and external validations, respectively.

**Conclusions:**

This research introduced an effective nomogram prediction model for predicting POD after radical hysterectomy for CC, providing a straightforward and visual method for individualized risk assessment.

## Introduction

Cervical cancer (CC) ranks as the fourth most common cancer and the fourth leading cause of cancer-related deaths among women worldwide (1). With the widespread adoption of CC screening technologies and human papillomavirus (HPV) vaccination, the incidence of CC has been declining annually in many developed countries (2). However, studies indicate that cancer incidence among the elderly population is 11 times higher than in other age groups (3). In 2022, China reported 150,700 new CC cases and nearly 60,000 deaths, with elderly women (aged over 60) accounting for 29.8% of cases. Elderly patients often present with later-stage diagnoses due to reluctance to seek medical care, fear of gynecological exams, and poorer overall health with multiple comorbidities, leading to worse prognoses (4). Based on the International Federation of Gynecology and Obstetrics (FIGO) guideline, radical hysterectomy with pelvic lymph node dissection is the primary curative treatment for early-stage CC (5), though minimally invasive approaches remain controversial. As a frequent complication in elderly surgical patients, postoperative delirium (POD) is associated with prolonged recovery, cognitive decline, and increased mortality (6). Recent studies have suggested that the development of POD is influenced by multiple factors, including age, comorbidities, and surgical stress (7, 8). One of the key mechanisms underlying POD may involve inflammation, which is triggered by both the surgical trauma and underlying cancer-related factors (9). Systemic inflammatory responses, characterized by the release of pro-inflammatory cytokines (such as IL-6, TNF-α), have been shown to contribute to neuroinflammation and affect brain function, leading to cognitive dysfunction post-surgery (10). This inflammatory response may also worsen the prognosis of elderly patients with cancer (11). Additionally, oxidative stress and blood-brain barrier disruption are other proposed mechanisms by which inflammation may induce or exacerbate POD in these patients (12).

Predicting POD enables early identification of high-risk patients, facilitating targeted interventions to reduce POD incidence (13). Given POD's significant impact on elderly patients—and its multifactorial pathophysiology involving age, comorbidities, surgical stress, and inflammation—there is a clear need for a comprehensive risk-prediction approach. In particular, systemic inflammatory activity and neuroinflammation have been implicated in POD. Accordingly, this study evaluates clinical, demographic, and laboratory variables—including inflammatory biomarkers and preoperative nutritional status—to identify independent predictors of POD. We hypothesize that these parameters (e.g., inflammatory indices, and nutrition metrics) can robustly predict POD and can be integrated into a nomogram to stratify individual risk, thereby enabling earlier, targeted prevention and ultimately improving postoperative outcomes.

## Materials and methods

A retrospective analysis was conducted on 253 elderly patients who underwent radical hysterectomy for cervical cancer (CC) at our hospital between 2021 and 2025. The criteria for inclusion were as follows: (1) a confirmed diagnosis of CC based on postoperative pathology, (2) American Society of Anesthesiologists (ASA) physical status classification I–III, (3) FIGO stage IA1 to IIA2, and (4) radical hysterectomy with pelvic lymph node dissection under general anesthesia. Patients were excluded if they (1) required emergency surgery, (2) had preoperative delirium, cognitive dysfunctions, psychiatry and neurological diseases, or (3) had incomplete clinical records. This study received approval from the institutional ethics committee (Approval No. JS 2023-076), and all participants provided written informed consent.

A standardized anesthesia protocol was implemented by the same surgical team. General anesthesia was administered via laryngeal mask airway (LMA) with the following induction regimen: sufentanil (0.5 μg/kg), propofol (2 mg/kg), and rocuronium bromide (0.6 mg/kg). Intraoperative maintenance was achieved with inhaled sevoflurane (2%−3% concentration) combined with continuous remifentanil infusion (0.25–1 μg/kg/min), adjusted in real-time based on hemodynamic parameters. Postoperative analgesia utilized an intravenous pump containing sufentanil (150 μg) and ondansetron (24 mg) in 100 ml saline, delivered at 2 ml/h.

### Data collection

Clinical data were retrospectively extracted from the electronic medical records of all enrolled patients. Demographic variables included age and body mass index (BMI). Clinical treatment-related information comprised the duration of surgery, surgical approach (laparoscopic or open), smoking status, presence of comorbidities such as hypertension and diabetes, ASA physical status classification, and intraoperative blood transfusion. Tumor-specific information encompassed FIGO stage, pathological type, CC differentiation grade, and lymph node metastasis status.

In addition, preoperative laboratory evaluations included measurements of liver and renal function, hematological indices, inflammatory markers, and nutritional status. Specifically, hemoglobin (Hb), white blood cell (WBC), alanine aminotransferase (ALT), aspartate aminotransferase (AST), serum creatinine, neutrophils, lymphocytes, and C-reactive protein (CRP) were recorded.

The principal study outcome was defined as the incidence of POD during the 7-day perioperative period. From postoperative days 1 to 7, the same anesthesiologist conducted daily POD evaluations using the internationally validated Confusion Assessment Method (CAM) (14). The diagnostic criteria are as follows: (1) acute onset or fluctuating mental status; (2) inattention; (3) disorganized thinking; and (4) altered level of consciousness. A diagnosis of POD requires the presence of criteria 1 and 2, plus either criterion 3 or 4.

The albumin-to-fibrinogen ratio (AFR) was derived by dividing fibrinogen by albumin levels, while neutrophil-to-lymphocyte ratio (NLR) and monocyte-to-lymphocyte ratio (MLR) with the same calculation method. The Geriatric Nutritional Risk Index (GNRI) was calculated through the formula: GNRI = [14.87 × serum albumin (g/L)] + [41.7 × (actual weight/ideal weight)], as previously validated (15). For the Controlling Nutritional Status (CONUT) score assessment (10), three biochemical markers were stratified (16): serum albumin: ≥3.5 mg/dl (0 points), 3.0–3.49 (2), 2.5–2.99 (4), <2.5 (6); Lymphocyte count: ≥1,600/mm^3^ (0), 1,200–1,599 (1), 800–1,199 (2), <800 (3); and total cholesterol: ≥180 mg/dl (0), 140–179 (1), 100–139 (2), <100 (3). The total CONUT score represented the sum of individual component scores.

### Statistical analysis

Data were analyzed using SPSS 26.0 and GraphPad 10.0, using *t*-test, Mann–Whitney *U*-test or Chi-square test. Independent predictors of POD were identified through univariate screening (*P* < 0.1) and multivariate logistic regression (*P* < 0.05), with multicollinearity assessment. The nomogram was developed in R 4.4.3 and evaluated via ROC analysis. Statistical significance was defined as *P* < 0.05.

## Results

A total of 253 patients were analyzed for the occurrence of postoperative delirium (POD) following surgery for CC, with 41 patients diagnosed with POD within postoperative 7 days (16.2%). Table 1 summarizes associations between clinical variables and POD. Patients who developed POD were notably older (*P* = 0.003) and had longer surgical durations (*P* = 0.035) compared to those without POD. No significant differences were observed for BMI, surgical approach, smoking status, hypertension, diabetes, ASA grade, FIGO stage, CC differentiation, pathological types, lymph node metastasis, or intraoperative blood transfusion (*P* > 0.05). Some laboratory parameters were also compared between the POD group and the non-POD group (Table 2). Significant differences were observed in CRP (*P* = 0.028), AFR (*P* = 0.009), NLR (*P* < 0.001), CONUT (*P* < 0.001), and GNRI (*P* < 0.001) between patients with or without POD. Other laboratory variables, including ALT, AST, creatinine, WBC, Hb, and MLR, showed no significant differences between the two groups (*P* > 0.05).

**Table 1:** Demographic and clinical variables associated with POD in elderly CC patients.

**Variables**	**POD**		***P*-value**
	**Yes (*****n*** = **41)**	**No (*****n*** = **212)**	
Age (year)	72.3 ± 3.7	70.4 ± 3.7	0.003^*^
BMI (kg/m^2^)	–	–	0.439
≥24.0	12 (29.3)	50 (23.6)	–
<24.0	29 (70.7)	162 (76.4)	–
Operation time (min)	173.3 ± 22.2	166.3 ± 18.8	0.035^*^
Route of surgery, *n* (%)	–	–	0.126
Laparoscopic	26 (63.4)	159 (75.0)	–
Open	15 (36.6)	53 (25.0)	–
Current smoker, *n* (%)	5 (12.2)	20 (9.4)	0.588
Comorbidities, *n* (%)	–	–	–
Hypertension	7 (17.1)	31 (14.6)	0.688
Diabetes	10 (24.4)	28 (13.2)	0.067
ASA grade, *n* (%)	–	–	0.446
I/II	32 (78.0)	176 (83.0)	–
III	9 (22.0)	36 (17.0)	–
FIGO stage, *n* (%)	–	–	0.666
IA1	8 (19.5)	53 (25.0)	–
IA2	2 (4.9)	5 (2.4)	–
IB1	12 (29.3)	63 (29.7)	–
IB2	3 (7.3)	10 (4.7)	–
IIA1	9 (22.0)	35 (16.5)	–
IIA2	7 (17.1)	46 (21.7)	–
Cervical cancer differentiation, *n* (%)	–	–	0.797
Poorly	7 (17.1)	40 (18.9)	–
Moderately	24 (58.5)	130 (61.3)	–
Well	10 (24.4)	42 (19.8)	–
Pathological type, *n* (%)	–	–	0.844
Squamous carcinoma	32 (78.0)	172 (81.1)	–
Adenocarcinoma	7 (17.1)	28 (13.2)	–
Adenosquamous carcinoma	2 (4.9)	12 (5.7)	–
Lymph-node metastasis, *n* (%)	8 (19.5)	38 (17.9)	0.809
Intraoperative blood transfusion, *n* (%)	4 (9.8)	45 (21.2)	0.129

POD, postoperative delirium; CC, cervical cancer; BMI, body mass index; ASA, American Society of Anesthesiologists; FIGO, International Federation of Gynecology and Obstetrics.

*P-value <0.05.

**Table 2:** Laboratory parameters associated with POD in elderly CC patients.

**Variables**	**POD**		***P*-value**
	**Yes (*****n*** = **41)**	**No (*****n*** = **212)**	
ALT (U/L)	23.5 ± 8.9	20.3 ± 10.4	0.067
AST (U/L)	21.0 ± 9.6	20.0 ± 7.0	0.434
Creatinine (mmol/L)	52.3 ± 5.7	53.5 ± 4.6	0.143
WBC (× 10^9^/L)	7.0 ± 2.0	7.1 ± 2.2	0.787
Hb (g/dl)	10.6 ± 0.9	10.5 ± 1.2	0.613
CRP, *n* (%)	–	–	0.028^*^
≥8 mg/L	12 (29.3)	32 (15.1)	–
<8 mg/L	29 (70.7)	180 (84.9)	–
AFR	9.1 ± 1.2	9.6 ± 1.1	0.009^*^
NLR	3.6 ± 0.5	3.3 ± 0.4	<0.001^*^
MLR	0.23 ± 0.06	0.22 ± 0.06	0.330
CONUT	4.2 ± 1.0	3.5 ± 1.1	<0.001^*^
GNRI	89.0 ± 8.4	95.2 ± 10.9	<0.001^*^

POD, postoperative delirium; CC, cervical cancer; ALT, alanine aminotransferase; AST, aspartate aminotransferase; WBC, white blood cell count; Hb, hemoglobin; CRP, C-reactive protein; AFR, albumin-to-fibrinogen ratio; NLR, neutrophil-to-lymphocyte ratio; MLR, monocyte-to-lymphocyte ratio; GNRI, Geriatric Nutritional Risk Index; CONUT, Controlling Nutritional Status.

*P value <0.05.

To ensure comprehensive risk factor identification, variables demonstrating marginal significance (*P* < 0.1) in preliminary analyses (Tables 1, 2) were incorporated into logistic regression modeling. Univariate analysis revealed seven candidate predictors significantly associated with POD (*P* < 0.05), including age, operative duration, CRP, AFR, NLR, CONUT, and GNRI (Table 3). Prior to multivariate modeling, collinearity diagnostics confirmed acceptable thresholds (all VIF <5, tolerance > 0.2). The final multivariate model established five independent determinants: advanced age (OR = 1.12, 95% CI: 1.01–1.24, *P* = 0.031), reduced AFR (OR = 0.69, 95% CI: 0.49–0.96, *P* = 0.029), elevated NLR (OR = 3.51, 95% CI: 1.71–7.21, *P* = 0.001), higher CONUT (OR = 1.81, 95% CI: 1.22–2.69, *P* = 0.003), and lower GNRI (OR = 0.94, 95% CI: 0.90–0.97, *P* = 0.001; Table 3).

**Table 3:** Univariate, multivariate logistic regression analysis for POD in elderly CC patients, along with multicollinearity test.

**Variables**	**Univariate**		**Multicollinearity test**		**Multivariate**	
	**OR (95% CI)**	***P*** **-value**	**Tolerance**	**VIF**	**OR (95% CI)**	***P*** **-value**
Age	1.14 (1.04–1.25)	0.004^*^	0.981	1.020	1.12 (1.01–1.24)	0.031^*^
Diabetes	2.12 (0.94–4.80)	0.071				
Operation time	1.02(1.00–1.04)	0.038^*^	0.975	1.026	1.02 (1.00–1.04)	0.069
ALT	1.03 (1.00–1.06)	0.073				
CRP (≥8.0 vs. <8.0 mg/L)	2.33 (1.08–5.03)	0.032^*^	0.982	1.019	2.44 (0.97–6.17)	0.059
AFR	0.67 (0.49–0.90)	0.009^*^	0.986	1.014	0.69 (0.49–0.96)	0.029^*^
NLR	4.26 (2.13–8.51)	<0.001^*^	0.964	1.037	3.51 (1.71–7.21)	0.001^*^
CONUT	1.74 (1.25–2.40)	0.001^*^	0.979	1.022	1.81 (1.22–2.69)	0.003^*^
GNRI	0.95 (0.91–0.98)	0.001^*^	0.987	1.013	0.94 (0.90–0.97)	0.001^*^

POD, postoperative delirium; CC, cervical cancer; ALT, alanine aminotransferase; CRP, C-reactive protein; AFR, albumin-to-fibrinogen ratio; NLR, neutrophil-to-lymphocyte ratio; GNRI, Geriatric Nutritional Risk Index; CONUT, Controlling Nutritional Status; VIF, variance inflation factor; OR, odds ratio; CI, confidence interval.

*P value <0.05.

**Figure 1:**
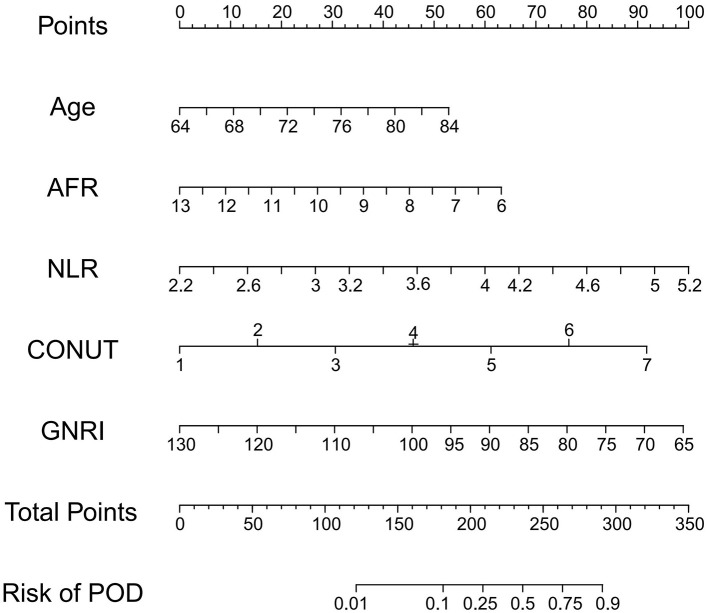
Nomogram model for POD in elderly CC patients. POD, postoperative delirium; CC, cervical cancer; AFR, albumin-to-fibrinogen ratio; NLR, neutrophil-to-lymphocyte ratio; GNRI, Geriatric Nutritional Risk Index; CONUT, Controlling Nutritional Status.

**Figure 2:**
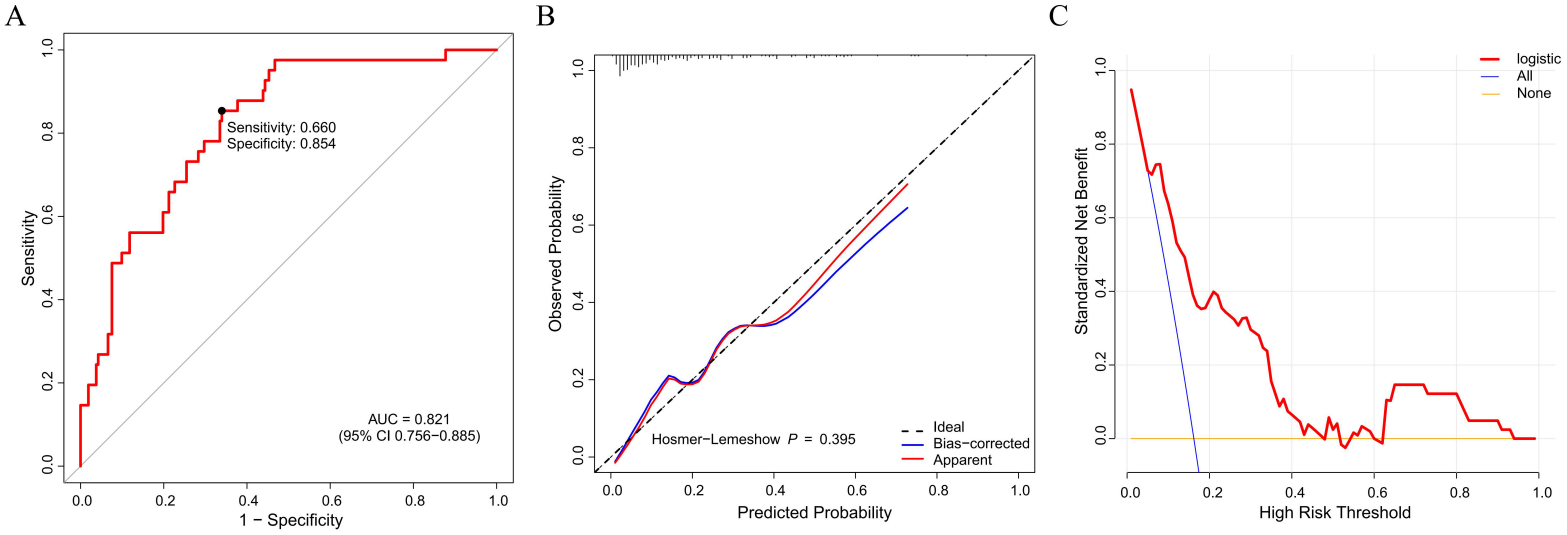
Nomogram model evaluation by ROC curve **(A)**, calibration **(B)**, and decision curve analysis **(C)**. ROC, receiver operating characteristic; AUC, area under the curve.

**Figure 3:**
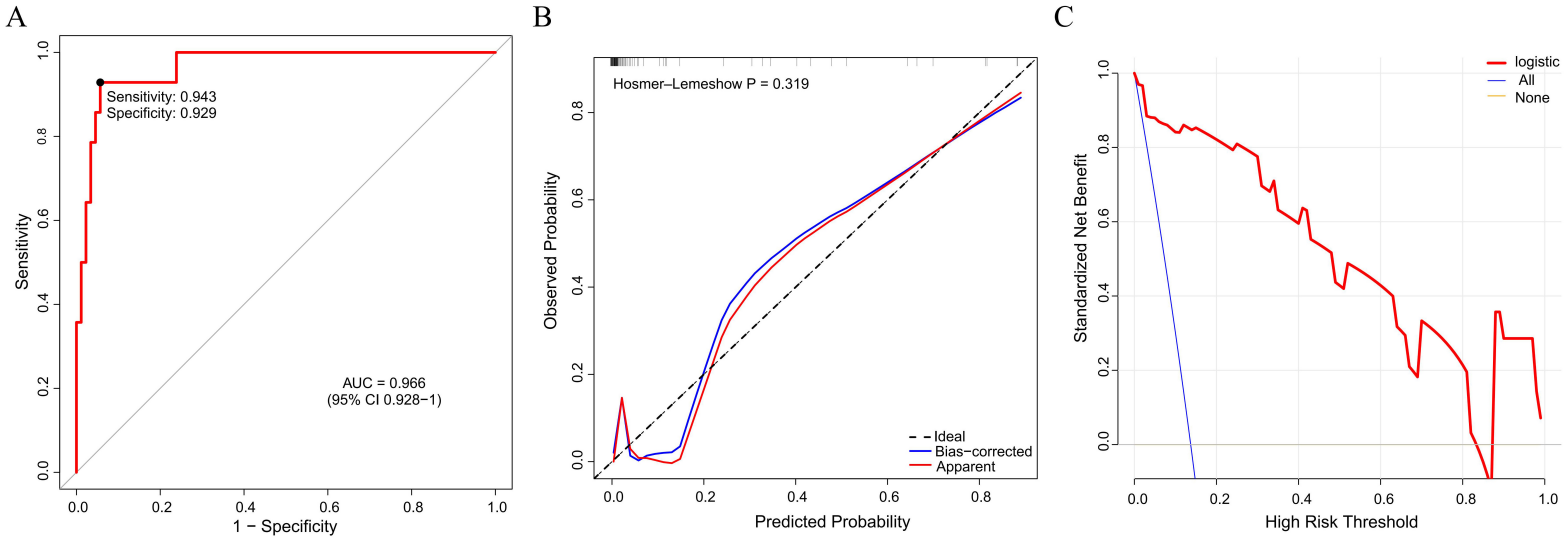
External validation of the nomogram model by ROC curve **(A)**, calibration **(B)**, and decision curve analysis **(C)**. ROC, receiver operating characteristic; AUC, area under the curve.

Based on the validated five independent predictors above (age, AFR, NLR, CONUT, and GNRI), we developed a nomogram to estimate the risk of POD (Figure 1). Each variable contributes a point score, and the total score corresponds to the predicted POD probability. Higher age, NLR, CONUT, and lower AFR and GNRI indicate increased risk. This tool offers a simple visual method for individualized POD risk prediction. Internal validation of the nomogram by ROC curve analysis demonstrated good discrimination with an AUC of 0.821 (95% CI: 0.756–0.885), a sensitivity of 0.660, and a specificity of 0.854 (Figure 2A). The calibration curve showed close agreement between predicted and observed probabilities, with a Hosmer–Lemeshow test *P* value of 0.395, indicating good model fit (Figure 2B). Decision curve analysis substantiated clinical applicability, with the nomogram outperforming extreme intervention strategies (treat-all/treat-none) across a wide range of threshold probabilities (Figure 2C). External validation using an independent cohort demonstrated excellent model performance, with an AUC of 0.966 (95% CI: 0.928–1), a sensitivity of 0.943, and a specificity of 0.929 (Figure 3A). The calibration (Figure 3B) and DCA (Figure 3C) curve analysis confirmed good model fit and a high clinical net benefit.

## Discussion

This investigation established and rigorously validated a preoperative risk stratification tool for POD in patients undergoing radical hysterectomy surgery for CC. Among the enrolled 253 patients, 16.2% developed POD within 7 days after surgery. Multivariate logistic regression analysis identified five independent predictors: age, AFR, NLR, CONUT, and GNRI. Based on these factors, we constructed a nomogram that demonstrated strong discriminatory power, good calibration, and clinical utility, based on multicenter external validation. The clinical significance of this study lies in addressing a critical knowledge gap: the lack of a simple, reliable, and specifically tailored predictive model for POD in CC patients. POD is a serious complication linked to prolonged hospitalization, increased costs, functional decline, and higher long-term mortality (17). While previous research has identified various risk factors for POD in general surgical populations, their applicability and relative importance in the specific context of CC surgery, which often involves a unique combination of oncological stress, pelvic dissection, and demographic factors, remained less clear. Our study moves beyond merely listing risk factors by integrating them into a quantifiable, visual tool (nomogram) that provides an individualized risk estimate, thereby facilitating personalized perioperative care. This is particularly important as it allows for the efficient allocation of limited resources for monitoring and preventive strategies toward the patients who need them most.

The observed age-dependent risk escalation corroborates existing neurobiological evidence regarding cholinergic system degeneration in geriatric populations (18). Age-related declines in cholinergic function, blood-brain barrier integrity, and mitochondrial efficiency may amplify susceptibility to surgical stressors (19). In our model, age remained an independent predictor of POD, supporting its importance in risk stratification. Notably, while operative time initially appeared significant, its effect was mediated by inflammatory/nutritional variables in multivariate analysis. This finding is clinically insightful, suggesting that the detrimental impact of prolonged surgery may be substantially mediated through its provocation of systemic inflammation and exacerbation of nutritional deficits, rather than being a risk factor in isolation. This underscores aging not merely as a chronological factor but as a marker of biological frailty, necessitating comprehensive preoperative assessment.

The inflammatory markers NLR and AFR also emerged as key predictors. Elevated NLR, an easily obtainable marker reflecting systemic inflammation and immune imbalance, has been linked to poor outcomes in various surgical and oncological settings (20), including POD (21). A high NLR indicates a proinflammatory state that may impair blood–brain barrier function and contribute to neuroinflammation (22), which are implicated in the pathogenesis of delirium. Conversely, albumin-to-fibrinogen ratio (AFR) serves as a composite marker of both nutritional status and inflammation (23). A lower AFR reflects hypoalbuminemia and/or hyperfibrinogenemia, both of which are associated with increased surgical stress and poorer prognosis (23). A recent systematic review and meta-analysis has indicated a close correlation analysis between AFR with POD in patients undergoing non-cardiac surgery (24), which was in accordance with our conclusions. Our results suggest that systemic inflammation plays a critical role in the development of POD and highlight the potential of these hematological parameters as early warning signals. The inclusion of both NLR and AFR in our model captures different facets of the inflammatory response, providing a more nuanced risk assessment than either marker alone.

Nutritional status, as measured by CONUT and GNRI scores, also showed significant associations with POD. Malnutrition has been recognized as a modifiable risk factor for delirium, as it can compromise immune function, delay recovery, and exacerbate stress responses (24). The CONUT score (derived from albumin, cholesterol, and lymphocyte) reflects both nutritional and immune status (25). Similarly, GNRI incorporates serum albumin and body weight and has been widely used as a prognostic marker in elderly or cancer patients (26). Malnutrition may exacerbate POD through impaired neurotransmitter synthesis and oxidative stress amplification (27, 28). Both indices were independently associated with POD in our study, emphasizing the relevance of nutritional assessment and optimization, for example, Enhanced Recovery After Surgery (ERAS) protocols, in perioperative care. The fact that both CONUT and GNRI retained independent significance alongside AFR (which also includes albumin) suggests that they capture distinct aspects of nutritional-immune frailty beyond serum albumin alone, such as cholesterol metabolism and body composition, offering a more comprehensive evaluation of the patient's baseline physiological reserve.

The nomogram developed in this study offers a practical tool for individualized risk prediction of POD in CC patients. It is user-friendly and integrates routinely available clinical and laboratory parameters, enabling clinicians to identify high-risk patients preoperatively. The model demonstrated strong internal and external validation performance, suggesting broad applicability. The calibration and DCA curve analysis confirmed clinical utility and good model fit, supporting its integration into preoperative checklists for targeted interventions—e.g., intensified monitoring, anti-inflammatory regimens, or nutritional supplementation in high-risk patients. The high net benefit on DCA, particularly in the clinically relevant threshold probability range, strongly justifies the potential implementation of this model in clinical practice. Specifically, its clinical impact lies in enabling a shift from a uniform postoperative approach to a risk-stratified, preemptive strategy. For patients identified as high-risk by the nomogram, clinicians could initiate targeted preventive measures pre- or intra-operatively. These could include intensified monitoring of cognitive status, tailored anti-inflammatory regimens, or preoperative nutritional supplementation to correct deficits highlighted by the CONUT and GNRI scores. This proactive approach holds the potential to reduce POD incidence, mitigate its associated complications, and improve overall perioperative outcomes.

This study importantly bridges a knowledge gap by synthesizing demographic, inflammatory, and nutritional parameters into a single predictive instrument. A key strength and justification for our approach is the demonstration that a model based on readily available preoperative variables can achieve high predictive accuracy, as evidenced by the exceptional performance on external validation. This suggests that the pathophysiological pathways represented by these variables—immunosenescence, systemic inflammation, and nutritional deficiency—are central to POD development in this patient population. We propose that these markers collectively depict a synergistic pathophysiology for POD: systemic inflammation (elevated NLR) promotes neuroinflammation and blood-brain barrier disruption, while nutritional deficits (reflected by CONUT/GNRI) impair neuronal resilience and neurotransmitter synthesis. The AFR acts as a nexus, integrating both inflammatory and nutritional stress into a single metric, thereby creating a vicious cycle of increased brain vulnerability.

In conclusion, we developed and validated a reliable nomogram incorporating age, AFR, NLR, CONUT, and GNRI to predict the risk of POD in elderly CC patients. This tool may aid clinicians in early identification of high-risk individuals, allowing timely preventive strategies and improved perioperative management.

This study has limitations that should be considered in interpreting the results. First, the retrospective, single-center design inherently risks selection bias and unmeasured confounding, which may compromise the internal validity of our findings and lead to overestimation of the predictors' effect sizes. Second, the limited generalizability beyond our specific institution and patient cohort means that the model's performance and the identified risk factors require external confirmation before they can be reliably applied in dissimilar clinical settings. Third, the use of single preoperative laboratory measurements, while practical, fails to account for physiological fluctuations, potentially reducing the stability and reproducibility of the individual biomarker scores in clinical practice. Moreover, whether the nomogram model retain its accuracy in more heterogeneous populations, including younger patients or those undergoing other oncologic surgeries remains unknown. Furthermore, the physiological mechanisms linking the nutritional-inflammatory markers to delirium pathogenesis were not explored in depth, leaving a gap in the biological rationale that future mechanistic studies should address. Finally, the assessment was confined to the immediate postoperative period, lacking data on medium- and long-term cognitive outcomes; consequently, the actual clinical utility of the nomogram in guiding targeted interventions to reduce POD incidence remains hypothetical, necessitating prospective interventional trials for validation.

## Data Availability

The raw data supporting the conclusions of this article will be made available by the authors, without undue reservation.
